# Investigation of the Molecular Profile of Granular Cell Tumours and Schwannomas of the Oral Cavity

**DOI:** 10.3390/dj10030038

**Published:** 2022-03-04

**Authors:** Benjamin Rogala, Zia Ali Khan, Linda Jackson-Boeters, Mark Roger Darling

**Affiliations:** Department of Pathology and Laboratory Medicine, Schulich School of Medicine and Dentistry, University of Western Ontario, London, ON N6A 5C1, Canada; brogala@uwo.ca (B.R.); zia.khan@schulich.uwo.ca (Z.A.K.); linda.jackson@schulich.uwo.ca (L.J.-B.)

**Keywords:** granular cell tumour, schwannoma, immunohistochemistry, RT-PCR, neural crest markers, APC markers

## Abstract

Granular cell tumours (GCTs) are rare submucosal lesions, thought to develop from Schwann cells, characterised by large polygonal cells with abundant lysosomes. The objectives of this study are to investigate whether GCTs have an antigen-presenting cell (APC) phenotype or a neural crest phenotype using immunohistochemistry and to compare expression profiles with Schwannomas. Immunoreactivity to CD68, HLA-DR, CD163, CD40 and CD11c (APC phenotype) and markers of neural crest cell (NCC) origin S100, SOX10, NSE and GAP43 in 23 cases of GCTs and 10 cases of Schwannomas were evaluated. RT-qPCR was used to identify a possible NCC developmental phenotype in 6 cases of GCTs. GAP43 was identified as a new NCC marker for GCTs, and some evidence was found for an APC phenotype from CD68 and HLA-DR immunoreactivity. RT-qPCR failed to identify an NCC developmental phenotype of GCTs, likely due to technical issues.

## 1. Introduction

Granular cell tumours (GCT) are relatively rare soft tissue lesions of which the aetiology has been a focus of interest for many years. They are thought to be derived from cells originating from the neural crest, specifically Schwann cells, based primarily on similarities between the immunohistochemical biomarker staining profile of GCT and schwannomas.

GCTs usually present as a solitary benign lesion, although multifocal and malignant cases are reported [1]. Approximately 50% of cases occur in the head and neck region, most commonly in the tongue, representing 23% to 28% of all lesions and 65% to 85% of intraoral lesions [1]. According to Allon et al., GCTs form 9.6% of benign tumours of the oral mucosa [2]. They present, most commonly, in the fourth to sixth decades, with a predilection for females over males of 2:1 [3]. Multiple lesions occur in 2–10% of cases [4].

GCTs are slow-growing indolent lesions, presenting as asymptomatic sub-epithelial firm round nodules [5]. In the oral cavity, the mean size at presentation is 2 cm, with the majority of lesions being treated before 3 cm in size [1]. GCTs are nondescript on imaging and, especially for larger lesions, may be interpreted as an invasive lesion on computerised tomography (CT) or magnetic resonance imaging (MRI) [6]. Tumours larger than 5 cm in diameter are suspicious for malignant transformation [7]. Narrow margin excision is the recommended treatment. Recurrence rates have been cited as 20% for incompletely excised lesions and at 2–8% of lesions with surgically clear margins, with recurrence likely the result of residual satellite cells/tumour extensions [8,9].

Grossly, GCTs are tan/pale-coloured, rubbery, and poorly circumscribed nodules without a capsule. Histologically, they are composed of larger-than-average polygonal- or lozenge-shaped cells of varying size, with abundant cytoplasmic eosinophilic granules and small eccentric nuclei. The granules are periodic acid Schiff (PAS)-positive and diastase-resistant [10] and stain positively with Luxol fast blue [1]. Tumour cells are arranged in clusters or sheets with interspersed thin/scant bands of connective tissue. The periphery of the tumour is poorly delineated, sometimes appearing to merge both with the overlying epithelium and with the underlying skeletal muscle, giving an infiltrative appearance. Additionally, there are often detached clusters of cells that can be found a distance from the bulk of the tumour, likely representing tumour extensions rather than budding lesions [1]. Reports of the presence of small nerves at the periphery of lesions suggest an association between GCTs and perineural cells and may imply occurrence following axonal injury [1]. Nearly 50% of cases show evidence of pseudoepitheliomatous hyperplasia, which may be mistaken for squamous cell carcinoma (SCC) by a pathologist unfamiliar with the lesion, confounded by the rarity of GCTs and the relatively high incidence of SCC [11,12].

GCTs were first described by Weber and Virchow in 1854 [6]. Abrikossoff theorised that they arose from myoblasts of the underlying skeletal muscle and named the tumour myoblastenmyome or myoblastoma [1]. Electron microscopy (EM) and immunohistochemical studies ruled out myogenous differentiation and were suggestive of a possible histiocytic origin, a possible common precursor to schwannomas, or undifferentiated mesenchymal precursors of fibroblasts that had phagocytised cellular debris [13,14,15,16,17]. Several studies supported neural sheath cell differentiation [9,16,17,18,19].

In contrast to the cell markers suggesting a neural origin, there has also been suggestions of an endomesenchymal origin. Gurzu found that GCTs expressed macrosialin (CD68), CD117 (c-kit), and RET [9]. GCTs have also been shown to express CD63, LC3 (microtubule-associated protein 1 light chain 3, a specific marker of autophagy) and antigen-presenting cell marker HLA-DR [20,21,22]. HLA-DR and CD68 immunoreactivity suggest an antigen-presenting cell (APC) phenotype for GCTs. Recently, novel inactivating mutations in ATP6AP1 and ATP6AP2 genes (associated with endosome acidification) have been identified in 72% of GCTs in a study by Pareja et al. [23]. It is theorised that changes in endosomal function result in altered cell signalling, giving GCTs oncogenic properties [23], providing evidence of a mutation that likely contributes to an APC phenotype of GCT. Additionally, considering the available evidence provided by IHC, it is not clear if GCTs are true neoplasms or reactive in nature.

HLA-DR, CD68, CD163, CD40 and CD11c are proteins expressed by APCs in executing their functions [24,25,26,27,28,29,30,31], while S100 protein, SOX10, NSE and GAP43 are proteins typically expressed by neural tissue, neural tumours and tissues of neural crest origin [32,33,34,35,36]. Growth associated protein 43 (GAP43) or neuromodulin is an intracellular growth-associated protein that has a critical role in guiding axonal growth and regulating neural cytoskeleton organisation and transduction of intra- and extra-cellular growth of neurites. It is inducible following nerve injury and plays a role in the new synapse formation of neural networks [35]. There are no prior reports investigating GAP43 expression in GCTs.

This study aims to differentiate an antigen-presenting cell (APC) phenotype from a neural tissue phenotype in GCTs using immunohistochemistry (HLA-DR, CD68, CD163, CD40 and CD11c for APC), and polymerase chain reaction and to compare this expression profile with schwannomas. Using RT-qPCR, we investigated RNA expression for nestin, SOX2, SOX9, SOX10, NF2, beta tubulin-III and glial fibrillary acidic protein (GFAP)—all proteins that are variably expressed by neural-crest-derived tissue and Schwann cells, in addition to CD68, beta tubulin-3 (a marker for neural tissue) and beta-actin as control [37,38,39,40,41,42,43].

## 2. Results

### 2.1. Demographics

The mean age for patients diagnosed with a biopsy-proven GCT was 41.4 ± 13.1. GCTs were most prevalent in the fourth to sixth decades of life, with 6 cases diagnosed in the fourth and fifth decades and 7 in the sixth decade. The mean age for patients with a biopsy-proven schwannoma was 25 ± 9. Lesions were most prevalent in the third decade of life. There were 14 cases of GCTs in females and 9 in males, giving a female:male ratio of 1.56:1. There were 8 cases of schwannomas in males and 2 in females, with a male:female ratio of 4:1. The most common location for GCTs was the dorsal tongue, with 16/23 lesions or 69.5% of cases. The mean diameter of the lesions was 10.1 mm, with a range of 5 to 20 mm. Lesions were treated by excisional biopsy, although in most cases, the deep margins were positive. Despite this, no recurrences or relapses were recorded. There was no obvious anatomical site predilection for schwannomas (Table 1).

### 2.2. Immunohistochemistry

**Figure 1:**
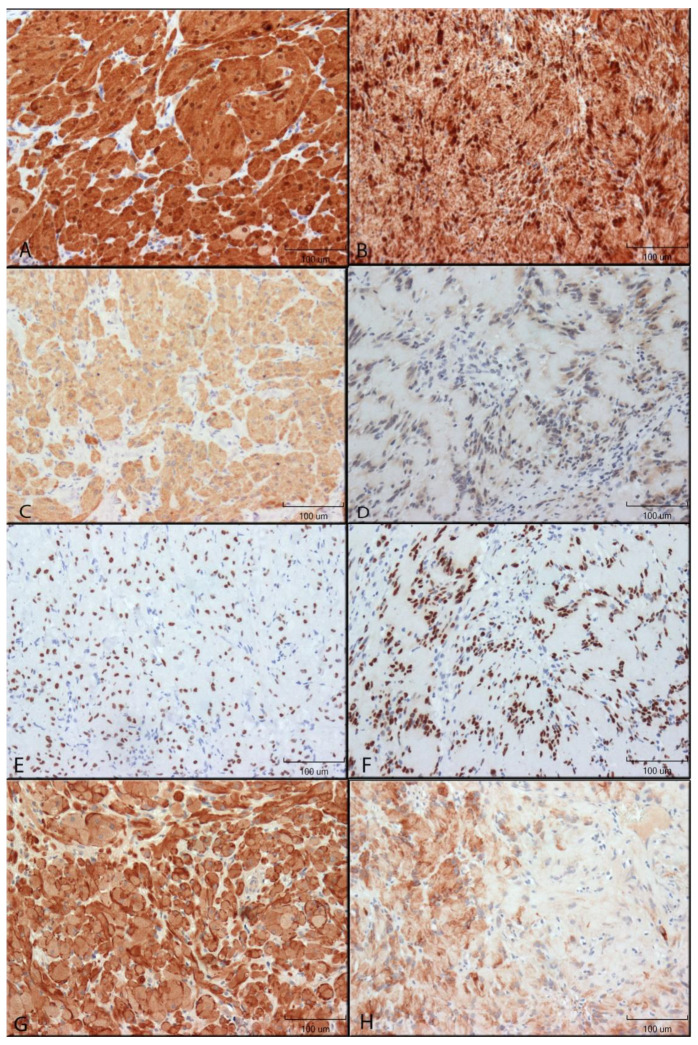
Immunohistochemical reactivity of neural markers in GCTs and Schwannomas. Immunoreactivity was detected by DAB (brown colour). Images were taken using 200× original magnification. Relative size shown by 100 µm scale bar. (**A**): S100 protein in GCT. (**B**): S100 protein in Schwannoma. (**C**): NSE in GCT. (**D**): NSE in Schwannoma. (**E**): SOX10 in GCT. (**F**): SOX10 Schwannoma. (**G**): GAP43 in GCT. (**H**): GAP43 in Schwannoma.

**Figure 2:**
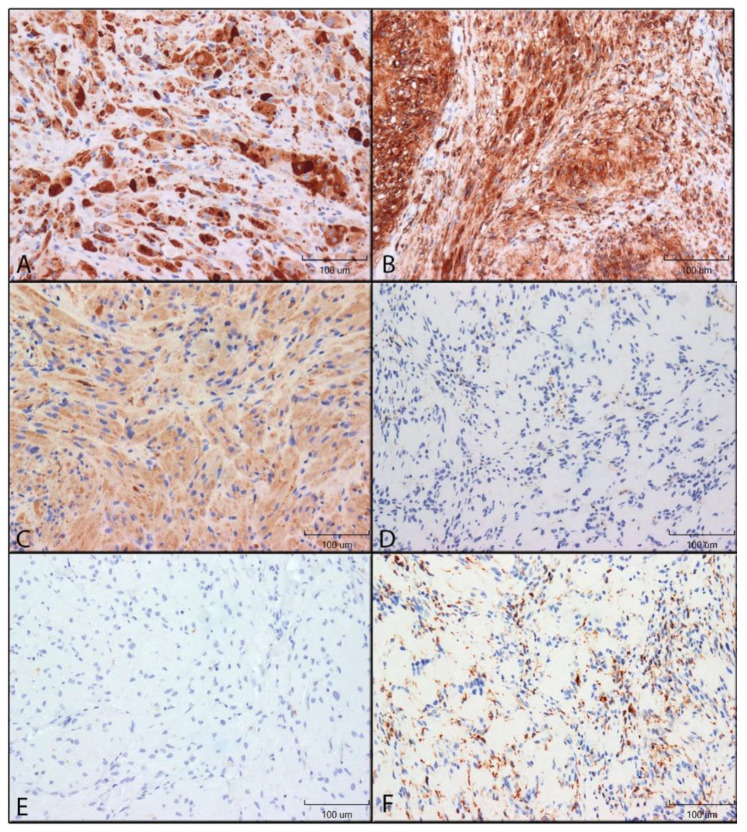
Immunohistochemical reactivity of antigen-presenting cell markers in GCTs and schwannomas. Immunoreactivity was detected by DAB (brown colour). Images were taken using 200× original magnification. (**A**): HLA-DR in GCT. (**B**): HLA-DR in schwannoma. (**C**): CD68 in GCT. (**D**): CD68 in schwannoma. (**E**): CD163 in GCT. (**F**): CD163 in schwannoma.

Immunohistochemical staining results are illustrated in Figure 1 and Figure 2.

**S100 staining:** S100 immunoreactivity was identified in both the cytoplasm and the nucleus of both GCTs and schwannomas, although nuclear staining was found to be greater than that of cytoplasm. In the spindle cells of schwannomas, the staining was punctate with bands of higher staining intensity in direct proximity to the nucleus, while cytoplasm staining of GCTs was more diffused. Nearly all granular cells of GCTs and spindle cells of schwannomas were found to stain, with little to no variability in the staining intensity (Figure 1A,B).

**NSE:** In GCTs, NSE staining was restricted to the cytoplasm, while Schwannomas had mostly cytoplasmic staining but also nuclear staining in many cells. Nearly all GCT granular cells were stained with high intensity. Most spindle cells (>50%) of schwannomas were positive (Figure 1C,D).

**SOX10:** SOX10 immunoreactivity was restricted to the nucleus of GCTs and Schwannomas and was of high intensity for all cells (Figure 1E,F).

**GAP43:** Both GCT and Schwannoma immunoreactivity was found to be restricted to the cytoplasm. For GCTs, two different cell staining patterns were identified: cells either stained mostly diffused with sparse punctate staining or cells stained mostly with a punctate pattern and minimal diffuse cytoplasmic staining. Within a given section, granular cells tended to either demonstrate more punctate or more diffused staining patterns.

Staining of the Schwannomas was considerably different from that of GCTs. All positive spindle cells demonstrated diffuse cytoplasmic staining, and a punctate pattern was not appreciated. Spindle cells found in the densely populated Antoni A areas were found to stain with high intensity. In contrast, the spindle cells of the Antoni B areas were either negative for GAP43 reactivity or stained with low to moderate intensity in less than 50% of the cells. This gave a patchy appearance to the Schwannoma GAP43 sections (Figure 1G,H).

**HLA-DR:** GCTs and schwannomas had similar immunoreactivity for HLA-DR. Staining was mostly restricted to the cytoplasm, with some nuclei appearing to stain positive for GCTs. Staining patterns were punctate on a diffuse background for both tumours. Nearly all granular cells of GCTs and spindle cells of Schwannomas were stained with high intensity, with minimal variability (Figure 2A,B).

**CD68:** CD68 immunoreactivity was restricted to the cytoplasm of both GCTs and schwannomas. GCT cells were stained with a light diffuse punctate pattern. Schwannoma staining was variable: one case was void of any appreciable staining for CD68 immunoreactivity, while the remaining 9 cases had low to moderate staining (Figure 2C,D).

**CD163:** Schwannomas were positive for CD63, with high intensity in all sections, while GCT immunoreactivity was absent. Schwannoma staining was punctate for all cells, and staining was restricted to the cytoplasm (Figure 2E,F).

**CD40 and CD11c:** Schwannomas and GCTs had no CD40 and CD11c immunoreactivity.

**Semiquantitative analysis using manual scoring:**Table 2 shows the cumulative scores for a percentage of cells stains and stain intensity, respectively, for both GCTs and schwannomas. A contingency table was constructed with the ordinal variable percentage cells staining as the x-variable and staining intensity as the y-variable. A linear-by-linear association test was completed. The *p*-value <0.0000000000000002 identifies that the two variables are almost perfectly correlated. We rejected the null hypothesis that there is no association between the variables’ percent cells stained and cell stain intensity. Assuming the correlation between variables, we chose to complete further analysis using only the ordinal variable for staining intensity and could extrapolate these findings to percent cells staining.

For each antibody used in this report, a chi-squared table was constructed using the ordinal variable (staining intensity) as the *x*-axis and nominal variable (cell type) as the *y*-axis. Given that we are performing multiple hypothesis tests, a Bonferroni correction was applied. CD40, CD11c, and S100 antibody scoring were identical between GCTs and schwannomas.

There was no statistical difference between the staining intensity for the antibodies SOX10 (*p* = 0.5), GAP43 (*p* = 0.03), and HLA-DR (*p* = 0.4). Significantly higher stain intensity was identified in GCTS for NSE (*p* = 0.000008) and CD68 (*p* = 0.0001). CD163 had significantly higher stain intensity in schwannomas (*p* = 0.00000007).

An additional Cochrane-Armitage test was completed to compare the cell staining intensity across all variables for GCTs and schwannomas. The staining intensities were compiled into a single chi-squared table. A significant difference was identified between the overall staining intensities for GCTs and schwannomas (*α* = 0.05, *p* = 0.000004).

No correlation was found between the expression profiles of specific markers and clinical characteristics such as the size or site of GCTs.

**Figure 3:**
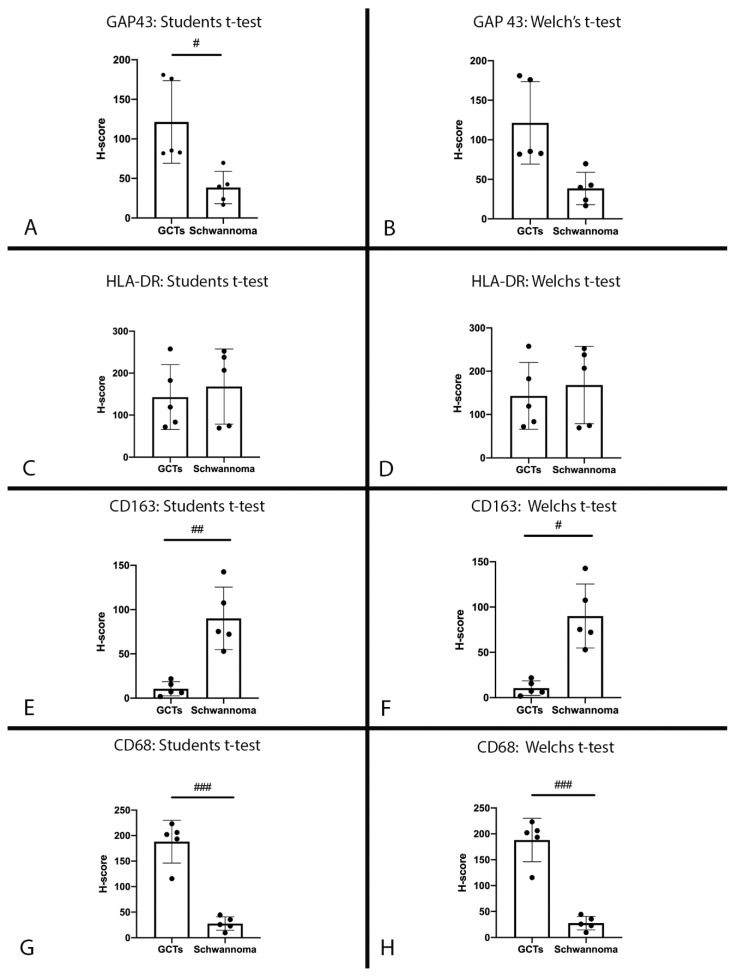
H-scores: H-score for GCTs and schwannomas for anti-GAP43 antibody. (**A**): Statistical comparison using Student’s *t*-test (*α* = 0.0125, *p* = 0.0108). (**B**): Statistical analysis using Welch’s *t*-test correction (*α* = 0.0125, *p* = 0.0201); # *p* ≤ 0.0125. H-score for GCTs and schwannomas for anti-HLA-DR antibody. (**C**): Statistical comparison using Student’s *t*-test (*α* = 0.0125, *p* = 0.646). (**D**): Statistical analysis using Welch’s *t*-test correction (*α* = 0.0125, *p* = 0.646). H-score for GCTs and schwannomas for anti-CD163 antibody. (**E**): Statistical comparison using Student’s *t*-test (*α* = 0.0125, *p* = 0.0012). (**F**): Statistical analysis using Welch’s *t*-test correction (*α* = 0.0125, *p* = 0.0062); # *p* ≤ 0.0125, ## *p* ≤ 0.005. H-score for GCTs and schwannomas for anti-CD68 antibody. (**G**): Statistical comparison using Student’s *t*-test (*α* = 0.0125, *p* < 0.0001). (**H**): Statistical analysis using Welch’s *t*-test correction (*α* = 0.0125, *p* = 0.0006); ### *p* ≤ 0.001.

**Q-score analysis obtained from Qupath Bioimage analysis:** QuPath was used to digitally identify cells with positive staining, which were then further stratified into low, medium and high staining intensities. It became apparent that the distribution of H-score may follow a non-Gaussian distribution; however, given the low case count, it was difficult to perform an analysis of variance. Therefore, both Student’s *t*-test for Gaussian distribution and Welch’s *t*-test for non-Gaussian distribution were performed. A Bonferroni correction was applied with *m* = 4 (4 tests), resulting in αbonferroni = 0.05/4 = 0.0125. Comparisons of H-score between GCT and schwannoma for GAP43, HLA-DR, CD68 and CD163 antibodies are demonstrated in Figure 3.

Digital scoring (H-score) was compared with the manual scoring intensity of cell staining using Pearson’s correlation coefficient and Pearson’s correlation for continuous variables (*t*-test). The results are summarised in Table 3. There was a near-perfect correlation between the manual scoring of cell staining intensity and the H-score derived from digital analysis using QuPath. A Bonferroni correction was not applied as the p-values for all analyses were extremely low.

### 2.3. Summary of Cq Value Obtained Using RT-qPCR

RT-qPCR was used to detect the presence of mRNA. Statistical analysis was not performed, and results are observational only. Six samples showed housekeeping gene amplification and were used for additional target gene measurement. Cases 18 and 23 showed the lowest Cq value, indicating a relatively high level of expression, while Cases 1, 3, 8 and 15 showed low expression.

CD68 transcript was identified in 5/6 specimens subjected to RT-qPCR. Case 1 did not show CD68 amplification, despite staining densely positive in all granular cells using IHC. The other cases had detectable CD68 expression.

With the exception of Case 23 and NTC, none of the other cases showed detectable SOX2, SOX9, SOX10, NF2 or nestin expression. Cases 18 and 23 were positive for BTub3 expression, while all others were negative. No cases showed SOX2 or GFAP mRNA presence (Table 4 and Table 5).

## 3. Discussion

The current understanding is that GCTs represent a tumour originating from Schwann cell lineage or differentiating into a Schwann cell phenotype [1], although a number of different tissues have been considered as an origin, including myoblasts [1], fibroblasts [13], histiocytes [14] and endomesenchymal cells [9]. A Schwann cell line of differentiation is supported by the similarity of their IHC profile for markers of neural crest cells and neuroectodermal origin, including SOX10, S100 and NSE.

GCTs and schwannomas have significantly different expressions of the lysosomal marker CD68, with GCTs staining strongly and diffusely and schwannomas staining weakly in only a small number of cells. CD68 expression supports a possible APC phenotype. Additional evidence that the granular appearance of GCTs results from lysosomes is supported by work identifying mutations of the gene coding for accessory proteins of H+-ATPase, ATP6AP1 and ATP6AP2, providing evidence of the abnormal lysosome content of the granular cells [23]. The finding of this genetic mutation of GCTs suggests that they are true tumours and not reactive lesions.

### 3.1. Protein Expression Supporting an NCC Origin of GCTs

Polyclonal S100 is one of the most commonly used antibodies employed by pathologists to assist with differentiating spindle cell lesions from NCC and non-NCC origins. The S100 polyclonal antibody used in this investigation has the highest specificity for S100B. There is copious evidence to suggest that neural and glial cells express a high level of S100 protein, particularly S100B, in their healthy state [44]. It is also generally accepted that all benign and most malignant tumours with NCC differentiation express S100 protein [44]. In contrast to the presumed specificity of S100B, a variety of tumours and cells originating from all germ layers can be induced to express S100 proteins, particularly S100A8, S100A9, S100A12 and S100B, as a result of oxidative stress and tissue inflammation [45]. The strong intensity of SOX10 immunoreactivity provides additional evidence of an NCC origin or NCC differentiation and is emerging as a more specific marker of NCC origin than S100. It is interesting to note that one case in this study was non-reactive for SOX10 immunoreactivity while staining with strong intensity in most cells for S100. Negative immunoreactivity to SOX10 was confirmed by repeating SOX10 IHC on a second section of this particular case. We considered excluding this case from our investigation but ultimately decided that the tissue was still interpretable for histopathology. The explanation for the lack of SOX10, in the context of the remainder of the staining profile of this case, could either be that during tumourigenesis, the tumour lost SOX10 expression or that the lack of SOX10 staining was a consequence of freezing during tissue transport. SOX10 has been shown to be expressed in virtually all BPNST; however, there are several reports identifying the absence of SOX10 staining in MPNST in 30% of tumours, which can be problematic for pathologists in diagnosing malignant spindle cell lesions [46]. We were unable to find any reports of either peripheral benign or malignant GCTs that did not express SOX10 but expressed S100 protein. Of note, central nervous system GCTs, which are presumed to develop from an astrocyte origin and have a virtually identical histological appearance to peripheral GCTs, were found to stain negatively for SOX10 [47]. In contrast, there are several case reports identifying GCTs found to be negative for S100 but positive for SOX10, both from the oral cavity and other anatomical sites. It is unclear if these tumours, referred to as primitive GCTs, are derived from mesenchyme of an NCC origin that differs from traditional GCTs or if these tumours have lost S100B expression [48]. While both GCTs and schwannomas stained positive for NSE, providing support of a NCC origin, an interesting finding of this investigation was that GCTs stained with higher intensity in a greater percentage of cells than schwannomas for NSE. This may indicate that GCTs have a higher metabolic demand than schwannomas as there are multiple reports that NSE is upregulated in both tumours derived from NCC and non-NCC-derived tumours during inflammation and hypoxic stress [34,49,50,51].

This report is the first to identify the GAP43 immunoreactivity of GCTs. GAP43 is a relatively newly discovered protein that appears to be a highly specific marker of neural and glial tissues. Its role in glial cells has yet to be determined; however, there is evidence to suggest it promotes the outgrowth of neurites during development and following nerve injury in growth cone formation [36]. It is interesting to note that GAP43 is expressed in repair Schwann cells and Schwann cell precursors but not in mature Schwann cells. Its expression also occurs late, following nerve injury at 4 weeks, which suggests that its expression occurs late in Wallerian degeneration following the clearance of myelin debris [7]. In contrast to S100 and SOX10, GAP43 expression is also retained by MPNST and has recently been suggested to have a higher sensitivity and specificity for NCC-derived spindle cell malignancies than either S100 or SOX10 [52]. Recent studies have identified GAP43 immunoreactivity in a variety of malignancies [53,54,55], calling into question the presumed specificity of GAP43 and suggesting that it has additional yet-to-be-determined roles in tumorigenesis. GAP43 immunoreactivity provides new evidence that GCTs originate from the NCC and provides the strongest evidence yet that GCTs likely develop from Schwann cells, suggesting that the granular cells of GCTs have a cell phenotype more similar to repair Schwann cells or Schwann cell precursors than mature Schwann cells.

Another finding in this report that has not been described is the architecture of schwannoma GAP43 staining. Immunoreactivity of the cell-rich Antoni A region was found to stain with high intensity in most spindle cells, while the Antoni B region was found to stain with low intensity in less than 50% of the spindle cells. Antoni B regions are generally thought to be degenerated regions of schwannomas associated with inflammation such as hyalinisation, fibrosis, mucin inclusion, thrombosis, and macrophage and lymphocyte infiltration [56,57]. Greater staining of the Antoni A regions would suggest that GAP43 immunoreactivity and protein expression decrease as the spindle cells of schwannomas begin to lose the architecture of Antoni A areas and transition to Antoni B areas. This would also suggest that the granular cells of GCTs are more similar to the spindle cells found in the Antoni A areas than the Antoni B areas.

### 3.2. Protein Expression Supporting an APC Phenotype

Collectively, the evidence from this investigation provides weak evidence of an APC phenotype for GCTs. At the onset of our investigation, the function of the increased lysosome content of the granular cells was unknown, and it was unclear if GCTs were reactive lesions or tumours. Consistent with other reports, this investigation identified strong staining intensity in the majority of granular cells for the CD68 antibody, indicating that the granules are lysosomes. CD68 is generally accepted as a cell marker for tissues with a phagocytic function, such as histiocytes, monocytes, giant cells, Kupffer cells, and osteoclasts, and its expression is thought to be regulated by a macrophage-specific promoter gene [26,27]. It had been theorised that lysosomes of GCTs may indicate a phagocytic function of GCTs following nerve injury. The presence of an abnormal number of lysosomes has now been explained by the work of Pareja et al., who have identified a novel mutation of the ATP6AP1 and ATP6AP2 genes coding V-ATPase accessory proteins, which have roles in endosome acidification and transport [23]. These findings provide clear genetic evidence to explain the intensity of CD68 immunoreactivity and confirm the theory that the accumulation of lysosomes is a result of altered lysosomal transport and function. Additional support of an APC phenotype for GCTs is the strong immunoreactivity for HLA-DR. HLA-DR expression is associated with APCs, including B lymphocytes, activated T lymphocytes and professional APCs (monocytes, macrophage and dendritic cells) [24]. We had theorised that in combination with CD68 immunoreactivity, HLA-DR immunoreactivity could suggest that GCTs may be reactive in nature and have a role in antigen presentation during the innate immune response. This theory is disproven by genetic evidence that the lysosomes of GCTs are non-functional and have negative reactivity for the costimulatory molecule CD40, suggesting that GCTs cannot participate in T-cell activation [58]. Given that GCTs are thought to have malignant potential, it is plausible that HLA-DR expression serves a role in GCTs analogous to HLA-DR expression by melanomas [59]. In melanomas, HLA-DR is thought to be associated with tumour-antigen presentation, tissue inflammation, and an immune response against tumour cells and is a positive prognostic factor for survival [59]. A third explanation is that the presence of HLA-DR immunoreactivity may suggest an epithelial–mesenchymal transformation, more indicative of tissue dedifferentiation and an APC phenotype rather than serving a role in antigen presentation. Finally, a fourth possible explanation is that expression of HLA-DR indicates tissue inflammation as HLA-DR expression has been reported in several different cell populations during tissue inflammatory responses [60,61,62]. HLA-DR immunoreactivity has not been previously described in schwannomas, and, significantly, all schwannomas in this study were positive for HLA-DR. While immunoreactivity of schwannomas to HLA-DR has not been reported, expression of HLA-DR has been reported in gliomas and neuroblastomas, where it is thought to be associated with increased tumour inflammation and worse patient outcomes [63].

The negative immunoreactivity of GCTs for CD163, CD40 and CD11c does not provide support for an APC phenotype of GCTs. An interesting finding that has not been previously reported was the strong intensity of schwannoma immunoreactivity for CD163. The upregulation of CD163 in schwannomas could possibly be explained by a cell phenotype of the spindle cells of schwannomas, similar to the phagocytic phenotype of repair Schwann cells, and also serve as a marker of an inflammatory process. In GCTs, we identified cells with a dendritic appearance that had reactivity to CD163. This supports inflammation within GCTs, which could result in HLA-DR expression, but does not provide support of an APC phenotype for GCTs.

### 3.3. Interpretation of RT-qPCR Data

Surprisingly, SOX10 mRNA was only detected in one case (Case 23) and non-reactive in 5/6 cases. The non-expression of SOX10 is in stark contrast to previously published literature regarding GCT immunoreactivity to SOX10 and is inconsistent with the IHC findings of this investigation. Given that findings from RT-qPCR are accepted to be more sensitive and specific than IHC findings, this result is perplexing. One possible theory is that SOX10 is a highly stable protein, such that very low levels of mRNA are required to maintain adequate SOX10 levels for cell function. The level of SOX10 mRNA could have been insufficient to be detected in 40 cycles of PCR. One other possible explanation is that SOX10 mRNA was more susceptible to degradation than either CD68 or beta-actin. Fragmented mRNA would still be detected using Qubit fluorescence, giving the impression that there was a sufficient concentration of RNA for further work. Alternatively, it could be a combination of both very low levels of SOX10 mRNA and mRNA degradation. Without positive SOX10 reactivity, it is challenging to make any further conclusions regarding the reactivity of nestin, SOX2, SOX9, SOX10, NF2 and GFAP.

### 3.4. Limitations of This Study

While digital analysis is likely more accurate than objective scoring methods and certainly seems to be better at detecting small differences in staining intensity, there are several issues with whole slide analysis using QuPath, related to manual settings and developing discriminatory algorithms for detection of cell boundaries, cytoplasm and nuclei.

In light of a strong body of evidence that GCTs are immunoreactive for SOX10, we elected to interpret the absence of SOX10 amplification as inadequate SOX10 mRNA quality despite PCR having a higher specificity and sensitivity than IHC techniques.

### 3.5. Future Work

It may be worthwhile investigating an NCC developmental phenotype of GCTs using an alternative RNA isolation protocol. More work needs to be done to explore the variety of lesions that can occur with a granular appearance. Specifically, it would be interesting to assess for mutations of lysosome formation, acidification and transportation using gene sequencing similar to the methods used to identify genetic mutations of ATP6AP1 and ATP6AP2 in GCTs.

## 4. Material and Methods

### 4.1. Case Selection

Formalin-fixed paraffin-embedded tissues (FFPE) of 23 serial/consecutive cases of benign GCTs and 10 cases of schwannoma 2006–2016 were retrieved from the archives of the Department of Pathology, Schulich School of Medicine and Dentistry, Western University. Malignant cases were not identified. Patient age, sex, and location of the lesion were recorded. Patient demographics are shown in Table 1.

### 4.2. Immunohistochemistry (IHC)

Neural markers S100, SOX10, NSE and GAP43 and APC markers HLA-DR, CD68, CD163, CD40 and CD11c were examined (Table 6); 5 μm tissue sections were obtained from hydrated FFPE blocks. The Dako Autostainer Link 48 was used for automated IHC staining of the S100, SOX10, NSE, HLA-DR, CD68 and CD163 antibodies. The slides also had a standard positive control tissue array, and at least one negative control section of each tumour type was utilised for each antibody.

Sections of GCTs and schwannomas, in addition to appropriate positive controls (schwannoma for GAP43 and tonsil for CD40 and CD11c) and negative case controls, were used in the procedure. Antigen retrieval was performed in citrate buffer pH6 using a decloaking chamber (Biocare Medical, Pacheco, CA, USA). Sections were cooled, washed in phosphate-buffered saline (PBS), placed in a humidified chamber and blocked for 30 min with 2.5% horse serum. Rabbit anti-human Gap43 antibody (1/5000 dilution, cat no. NB300-143 Bio-Techne Canada Corporation, Oakville, ON, Canada), rabbit anti-human CD40 antibody (1/1000 dilution, Cat no. ab13545 Abcam, Toronto, ON, Canada) and rabbit anti-human CD11c (1/300 dilution, Cat no. ab52632 Abcam, Toronto, ON, Canada) were applied. The primary antibodies were incubated overnight at 4 °C with a negative control.

On day 2, the sections were washed and then incubated with a secondary antibody. GAP43 and CD40 slides were incubated with immPRESS anti-rabbit IgG (Cat No. VECTMP540150 MJS Biolynx, Brockville, ON, Canada) for 30 min at room temperature. CD11c sections were incubated with Avidin-Biotin Complex VECTASTAIN Elite ABC-HRP Kit (Ready-to-Use) (Cat no. PK-7200, Vector Laboratories, Burlington, ON, Canada) at room temperature for 30 min. To visualise the staining, sections were incubated with a 3,3′-diaminobenzidine (DAB) peroxidase substrate kit (Cat no VECTSK4100 MJS Biolynx, Brockville, ON, Canada). The sections were counterstained with Harris hematoxylin (Leica Biosystems Inc., Concord, ON, Canada).

### 4.3. Evaluation of Immunostaining

All slides were examined under light microscopy and scored according to the semiquantitative methods described below. In addition to manual scoring, 5 randomly selected cases of both GCTs and schwannomas for the antibodies GAP43, CD68, HLA-DR, CD163 were subjected to digital analysis using QuPath, an open software platform for bioimage analysis, a free open source digital bioimage analysis software that has been validated for its accuracy and reproducibility of results [64,65], available at https://qupath.github.io/ (accessed on 1 June 2020).

#### 4.3.1. Manual IHC Scoring

Each IHC slide was evaluated under light microscopy, utilising the semiquantitative analysis described below. Only the large polygonal cells that matched the histological description of the granular cells of GCTs were assessed. For schwannomas, analysis was restricted to tumour cells within the capsule. The scoring criteria consisted of an objective analysis of the average number of cells that stained positive within the representative high-power section. Staining intensity was subjectively scored relative to the appropriate positive controls. The scoring system used was as follows: for staining intensity: 0 = absent; 1 = weak; 2 = moderate; 3 = high; and for percentage of cells stained: 0 = 0%; 1 = 0–50%; 2 > 50%.

#### 4.3.2. IHC Digital Analysis

Five cases each of GCTs and schwannomas underwent high-resolution whole slide imaging using an Aperio slide scanner for the antibodies GAP43, HLA-DR, CD163 and CD68. Images were then uploaded to QuPath (v0.1.2). Images stained with the same IHC antibody (5 cases of GCT and 5 cases of schwannoma) were grouped to create a multi-slide project). For each of the four multi-slide projects, the same workflow was followed. Automated cell detection was completed by identifying cell nuclei via the hematoxylin optical density and nucleus size parameters. Positive cell detection was determined by the mean DAB optical density of the cell. A detection classifier was trained by adjusting the DAB optical density classification for low, medium and high intensities after visually selecting cells that had objectively low, medium and high staining intensities as a reference. Data was reported as a percentage of cells detected and also as an H-score. H-scores range from 0–300 and are calculated by 3× percentage of strong staining + 2× percentage of moderate staining + 1× percentage of weak staining (total ranging from 0 to 300).

### 4.4. Protocol for RT-qPCR

RNA was extracted from GCT FFPE tissue blocks and cDNA synthesised for the detection of CD68, nestin, SOX2, SOX9, SOX10, NF2, GFAP, beta-actin and B-Tub3.

#### 4.4.1. Tissue Preparation

Eleven FFPE blocks were assessed to have sufficient tumour volume to proceed with tissue isolation prior to RNA extraction. The corresponding H&E slide was examined under light microscopy to identify a region of the tumour that demonstrated typical features of GCTs, and non-tumour cells were excluded. A 1 mm punch biopsy of the FFPE block was obtained at a depth of 1 mm. The tissue plug was then placed into 1.5 mL collection tubes; 5 μm tissue sections of each case were obtained from hydrated FFPE blocks to verify that the site of the punch biopsy was representative of the tumour. H&E staining of these sections was completed, and the sections were visualised under the microscope, confirming the accuracy of the tissue punches were restricted to tumour tissue in all 11 cases.

#### 4.4.2. RNA Isolation

The specimens were deparaffinised, and RNA isolation was completed using the High Pure FFPE RNA Micro Kit (Roche Applied Sciences, Mannheim, Germany, Catalogue number: 04823125001). Tissue lysis buffer and 10% sodium dodecyl sulfate were added to the tissue plugs, followed by protein kinase K solution. The tubes were then agitated and incubated at 55 °C for 3 h. The cell lysate was extracted and placed into an RNA-binding spin column. Each tube was then spun on a centrifuge, and the flow-through was discarded. DNase solution was added, and the tubes were allowed to incubate for 15 min at room temperature. The RNA was then purified by rinsing the RNA with a series of buffer solutions, centrifuging at 8000× *g* for 5 min and discarding the supernatant each time. Elution buffer was added and allowed to incubate at room temperature for 1 min. The RNA spin column was then centrifuged at 8000× *g* for 1 min. The RNA eluate was collected and used for RNA quantification.

#### 4.4.3. RNA Quantification

RNA concentration of the RNA eluate was measured using the Qubit Quan-iT RNA BR assay kit (Thermo Fisher Scientific Carlsbas, CA, USA, catalogue number Q10210) and a Qubit fluorometer. Qubit RNA BR reagent was diluted to 1:200 in Qubit RNA BR buffer; 198 µL of prepared Qubit working solution and 2 µL of RNA eluent were added to clear 0.5 mL tubes. An additional 2 tubes were prepared by adding 10 µL Qubit standards to 190 µL of working solution and were used to calibrate the fluorometer to the standards. The concentration of RNA was then referenced to the prepared standards. RNA concentration in µg/mL was determined using the formula: RNA concentration = QF x (200/x)

#### 4.4.4. cDNA Synthesis and RT-qPCR

In total, 6 of 11 samples yielded sufficient RNA to proceed with complementary DNA (cDNA) synthesis. Isolated RNA was transcribed to cDNA using the iScript cDNA Synthesis Kit (Bio-Rad Laboratories, Inc., Hercules, CA, USA, catalogue number: 1708890). For each sample, 20 µL of cDNA mixture was prepared (4 µL 5x iScript reaction mix containing reverse transcriptase, 100 ng RNA and balance of the volume as nuclease-free H_2_O). RT-qPCR reactions were carried out in 96-well arrays (Hard-Shell^®^ Low-Profile Thin-Wall 96-Well Skirted PCR Plates, Bio-Rad, HSP-9601). Each well contained 10 µL RT^2^ SYBR Green qPCR Mastermix (Bio-Rad, 330501), 2 µL primer, 1 µL cDNA mix and 7 µL nuclease-free water. A no-template control (NTC) was used, which contained 8 µL of nuclease-free water and no cDNA. The array was constructed with case number as the *y*-axis and RT-qPCR was completed over a total of 40 cycles of amplification in CFX Connect (Bio-Rad Laboratories, Mississauga, ON, Canada). The assay information is shown in Table 7.

### 4.5. Statistical Analysis

Given that percent cells stained and stain intensity are both ordinal variables, a linear-by-linear association test was completed to assess the correlation of the two variables between the pooled GCT and schwannoma data, resulting in a near-perfect correlation. Due to the high level of correlation, only stain intensity was used for further statistical analysis, and results comparing staining intensity between GCTs and schwannomas could be extrapolated to the percentage of cells staining. A Cochrane-Armitage test for trend, using a generalised chi-squared test, was used to compare the overall staining intensities for GCTs versus schwannomas and for the 9 IHC antibodies used with the ordered nominal variable (intensity) and a non-ordered nominal variable cell type. As multiple hypotheses were applied to the same data set, a Bonferroni correction was applied. Three of the IHC stains, S100, CD40 and CD11c, had no variability within their data set and were of equal values between GCTs and schwannomas, so they were not used when calculating the Bonferroni correction with *m* = 6 (αbonferroni = 0.5/6).

Using the data generated by digital analysis, Student’s *t*-test and Welch’s *t*-test correction were performed to assess for differences between the H-scores of GCTs and schwannomas. Again, a Bonferroni correction was used with m = 4 (Corrected.αbonferroni 0.5/4). A Pearson correlation coefficient test was completed, comparing the H-scores and the staining intensity for each individual IHC antibody.

## 5. Conclusions

This study provides new evidence of an NCC origin or path for GCTs, probably of Schwann cell lineage, as supported by IHC immunoreactivity of GAP43 (significantly) as well as S100 protein, SOX10 and NSE. The expression of HLA-DR and CD68 supports the induction of an APC phenotype in a nerve sheath tumour. Because of the strong evidence that GCT tumourigenesis is along the NCC lineage, we suggest that GCTs be added to the peripheral nerve sheath tumour classification system. The term “granular cell nerve sheath tumour” might be more descriptive of this relatively rare lesion, and an appropriate immunophenotypic and molecular panel (including GAP43) should be implemented in the diagnosis of GCTs.

## Figures and Tables

**Table 1:** Demographics and site of biopsy.

Granular Cell Tumours			
**Case No.**	**Age**	**Sex**	**Site**
1	41	Female	midright dorsum of tongue
2	33	Female	dorsum of tongue
3	15	Female	dorsum of tongue
4	43	Male	dorsum of tongue
5	20	female	right tongue
6	36	Male	dorsum of tongue
7	48	female	left posterior hard palate
8	50	Male	dorsum of tongue
9	37	female	left lateral border of tongue
10	55	female	right palatal mucosa
11	39	female	dorsum of tongue
12	21	female	right dorsum of tongue
13	52	male	right lateral border of tongue
14	48	male	right dorsum of tongue
15	46	male	right dorsum of tongue
16	35	female	right dorsum of tongue
17	63	female	left dorsum of tongue
18	55	female	left dorsum of tongue
19	37	male	dorsum of tongue
20	21	female	right lateral border of tongue
21	56	male	dorsum of tongue
22	58	male	right lateral ventral tongue
23	43	Female	left dorsal tongue
**Schwannomas**			
**Case No.**	**Age**	**Sex**	**Site**
1	28	Male	buccal mucosa 37
2	21	Male	left dorsum of tongue
3	23	Male	right tip of tongue
4	41	Male	left buccal mucosa
5	16	Female	*** oral cavity
6	37	Female	lower lip mucosa
7	22	Male	lower lip mucosa
8	15	Male	left buccal vestibule
9	16	Male	dorsum of tongue
10	31	Male	Upper lip

*** Site of biopsy was unspecified, anatomically from the oral cavity.

**Table 2:** Mean DAB stain intensity and percentage of cells staining in GCTs and schwannomas.

IHC Antibody		Absent	Low	Medium	High	0 (0%)	1 (>0≤50%)	2 (>50%)
S100	GCT	-	-	-	23	-	-	23
	Schwannoma	-	-	-	10	-	-	10
NSE	GCT	-	-	3	20	-	-	23
	Schwannoma	-	4	6	-	-	-	10
SOX10	GCT	1	-	-	22	1	-	22
	Schwannoma	-	-	-	10	-	-	10
GAP43	GCT	-	-	2	21	-	-	23
	Schwannoma	-	-	4	6	-	3	7
HLA-DR	GCT	-	-	6	17	-	-	23
	Schwannoma	-	-	4	6	-	-	10
CD68	GCT	-	-	17	6	-	-	23
	Schwannoma	1	6	3	-	1	6	3
CD163	GCT *	22 *	-	-	-	22 *	-	-
	Schwannoma	-	-	1	9	-	-	10
CD40	GCT	23	-	-		23	-	-
	Schwannoma	10	-	-		10	-	-
CD11c	GCT	23	-	-		23	-	-
	Schwannoma	10	-	-		10	-	-

* Case 2 of GCT did not have a section for CD163 IHC.

**Table 3:** Summary of the comparison of cell stain intensity using manual semiquantitative scoring and H-score derived from QuPath.

Antibody	Correlation (r)	95% CI	*p*-Value
All tumours	0.906	0.827–0.949	0.000000000000001
GCTs only	0.903	0.767–0.961	0.00000005
Schwannomas only	0.9	0.759–0.960	0.00000007
GAP43	0.92	0.689–0.981	0.0002
HLA-DR	0.961	0.841–0.991	0.000009
CD68	0.989	0.951–0.997	0.00000007
CD163	0.979	0.911–0.979	0.0000008

**Table 4:** Summary of RNA concentration and B-actin and CD68 amplification cycles.

Case	RNA Concentration (µg/mL)	B-Actin Reactivity (Cq Value)	CD68 Reactivity (Cq Value)
1	6.5	34.70	-
3	17.4	37.38	37.15
6	4.24 *		
7	2.88 *		
8	5.7	36.94	35.96
15	7.4	34.53	37.12
16	Low *		
17	2.2 *		
18	10	29.71	32.53
22	4.16 *		
23	6.5	28.89	25.60
NTC		-	-

* = RNA concentration insufficient to proceed with RT-qPCR. - = no amplification.

**Table 5:** Summary of Cq values for Case 23.

Gene	Cq Value
CD68	29.60
Nestin	39.02
SOX2	-
SOX9	36.18
SOX10	38.01
NF2	37.14
GFAP	-
Beta Actin	28.89
Beta TUB3	36.72

- = no amplification.

**Table 6:** Summary of the antibodies used for immunohistochemistry of GCTs and schwannomas.

Antibody	Manufacture/Cat Number	Antibody Type	Constituent Tissue and Labelling Targets	Dilution
S100	Dako, Santa Clara CA IR50461-2	Rabbit polyclonal	Neural tissues, S100B (strong) S100A1 and S100A6 (weak)	Automated
NSE	Dako, Santa Clara CA IR61261-2	Mouse monoclonal	Neural tissues, γ-enolase subunit	Automated
SOX10	Santa Cruz Biotech, Dallas TX, sc-365692	Mouse monoclonal	Neural tissues, SOX10 transcription factor	Automated
GAP43	Bio-Techne Canada, Oakville On, NB300-143	Rabbit polyclonal	Regenerating neural tissues/growth cones, GAP43 intracellular growth protein/membrane protein	1/5000
HLA-DR	Dako, Santa Clara CA MO74601	Mouse monoclonal	APCs and lymphocytes, Alpha-chain of HLA-DR cell surface receptor	Automated
CD68	Dako, Santa Clara CA GA61361-2	Mouse monoclonal	Macrophage, lysosomal-associated membrane proteins	Automated
CD163	Vector laboratories, Burlingame CA VP-C374	Mouse monoclonal	Macrophage, hemoglobin-scavenger receptor	Automated
CD40	Abcam, Toronto ON, ab13545	Rabbit polyclonal	APCs, cell surface innate immune response costimulatory protein	1/1000
CD11c	Abcam, Toronto ON, ab52632	Rabbit monoclonal	APCs, cell surface fibrinogen receptor	1/300

**Table 7:** Summary of primers employed in RT-qPCR.

Gene Assay	Vendor	Cat Number	Amplicon Length	Exon-Spanning
CD68	QIAGEN	QT00037184	73 bp	Y
NESTIN	QIAGEN	QT01015301	75 bp	Y
SOX2	QIAGEN	QT00237601	64 bp	N
SOX9	QIAGEN	QT00001498	111 bp	Y
SOX10	QIAGEN	QT01670326	145 bp	Y
NF2	QIAGEN	QT00030191	148 bp	Y
GFAP	QIAGEN	QT00081151	96 bp	Y
Beta-Actin	QIAGEN	QT01680476	104 bp	Y
Beta-TUB3	QIAGEN	QT00083713	78 bp	Y

## Data Availability

The data presented in this study are available on request from the corresponding author. The data are not publicly available due to storage on office computer drive, as goberned by guidelines of the Review and Ethics Board of Western University.
